# Pure Red Cell Aplasia Associated With Thymoma

**DOI:** 10.7759/cureus.39387

**Published:** 2023-05-23

**Authors:** Ramesh Thangatorai

**Affiliations:** 1 General Medicine, Sultan Ismail Hospital, Johor Bahru, MYS

**Keywords:** cyclosporine a, malignant thymoma, symptomatic anemia, red cell aplasia, thymoma-associated pure red cell aplasia

## Abstract

A 27-year-old male with thymoma presented with recurrent severe anemia which required multiple transfusions. Bone marrow biopsy showed pure red cell aplasia with normal other cell lineages. He was diagnosed with pure red cell aplasia associated with thymoma and treatment with cyclosporine A was begun. After 12 weeks of treatment, his hemoglobin improved to 11.3 g/dL and the patient remained transfusion independent.

## Introduction

Thymoma is a relatively rare neoplasm that represents 47% of mediastinal tumors [1]. There is no known risk factor for thymoma. Thymomas can be classified as type A thymoma, AB thymoma, type B thymoma, micronodular thymoma with lymphoid stroma, and metaplastic thymoma depending on the histologic features. A patient with thymoma can present as an asymptomatic incidental finding on imaging, or they can become symptomatic due to local compressive effects of the mass. Patients can also rarely present with paraneoplastic syndromes associated with thymoma. Thymoma-associated paraneoplastic syndrome varies from neurological to hematological manifestation. We present a patient with hematological paraneoplastic syndrome associated with thymoma.

## Case presentation

A 27-year-old male with a diagnosis of anterior mediastinal thymoma presented with recurrent palpitations, lethargy, and exertional dyspnea, which required multiple admissions and investigations. He was first diagnosed with thymoma in 2017 when he presented with dyspnea and a subsequent CT thorax revealed a mediastinal mass with extension into left pleural space. An ultrasound-guided biopsy of the pleural mass was reported as thymoma, likely type B. He was reviewed by a cardiothoracic surgeon and his tumor was deemed inoperable, thus he was given chemotherapy and radiotherapy. In early 2021, four years after the diagnosis of thymoma, he developed recurrent symptomatic anemia.

On our review, he had hemoglobin of 3.6 g/dL, with normal white cell counts and platelet counts (Table 1). His reticulocyte counts were low, at 0.6×10^3^/UL. We performed first-line investigations for his anemia, which is shown in Table 1. He had normal B12/folate/iron levels. His Coombs test was normal, and thyroid-stimulating hormone was within normal limits. His peripheral blood film showed severe normochromic normocytic anemia with normal white cells and platelets. Esophagogastroduodenoscopy was done prior to medical consultation and did not reveal any bleeding foci in the upper gastric region. Physical examination revealed a pale average-built male, with no jaundice, no palpable cervical lymph nodes, nor any stigmata of nutritional deficiencies. He had no hepatosplenomegaly.

**Table 1:** Laboratory data from admission till follow-up posttreatment.

Tests	Range	Dates				
		03/01/21	03/02/2021	09/03/21	21/03/21	12/04/21
Hemoglobin (g/dL)	13-18	3.6	6.3	8.4	7.0	11.3
Mean corpuscular volume (fL)	80-100	81.2	81.8	84	84	83
Mean corpuscular hemoglobin concentration (g/dL)	27-32	33.5	33.2	33	33	32
White blood cell (×10^3^/uL)	4-11	4.6	5.7	7.4	6.8	8.0
Platelet (×10^3^/uL)	150-300	272	327	236	348	390
Reticulocytes (×10^3^/uL)	20-80	0.6	2.3	-	-	98.7
Folate (nmol/L)	>4	54.36	-	-	-	-
B12 (pmol/L)	180-1000	561	-	-	-	-
Transferrin saturation (%)	20-50	85	-	-	-	-
Iron (mcmol/L)	11-35	41.3	-	-	-	-
Coombs test	-	Negative	-	-	-	-
Thyroid-stimulating hormone (mIU/L)	0.4-4.0	2.02	-	-	-	-
Urea (mmol/L)	2.5-7.8	1.8	-	-	-	2.4
Creatinine (mmol/L)	59-104	67	-	-	-	76
Aspartate transaminase (U/L)	1-45	24	-	-	-	-
Alanine transaminase (U/L)	<41	38	-	-	-	-

**Figure 1:**
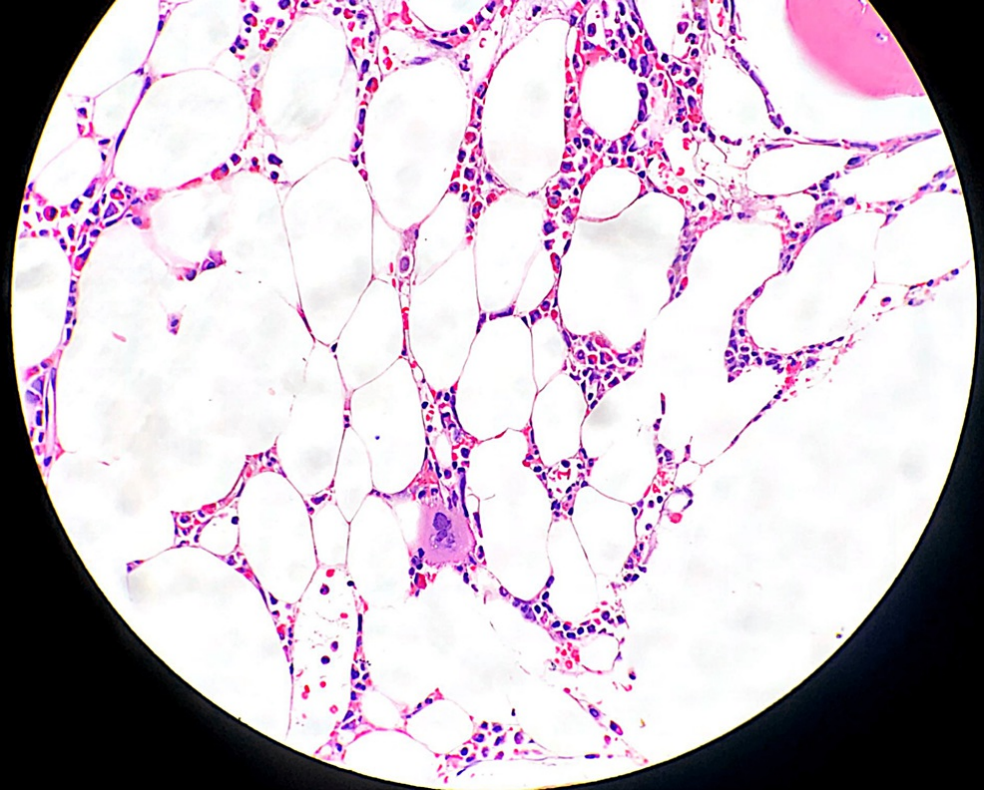
Bone marrow trephine showing paucity of erythroid precursors.

We then proceeded with diagnostic bone marrow trephine biopsy. His trephine biopsy is shown in Figure 1. It showed markedly hypocellular marrow with a marked reduction of erythropoiesis along with a lack of erythroid islands and maturing erythroblasts. Otherwise, the granulopoiesis and megakaryopoiesis were adequate with normal morphology, confirming our diagnosis of pure red cell aplasia.

Once the diagnosis of pure red cell aplasia was made, we referred this patient to a cardiothoracic surgeon for the removal of thymoma. Given that the tumor was inoperable due to extensive involvement of adjacent structures, we then began this patient on Tab. cyclosporine 150 mg BD (5 mg/kg/day) on March 9, 2021. We followed up on this patient for six months, and his hemoglobin level persistently improved at six-month follow-up.

## Discussion

There are many paraneoplastic syndromes associated with thymoma. Common paraneoplastic syndromes are myasthenia gravis, pure red cell aplasia, lichen planus, Good syndrome, and limbic encephalitis [2]. This patient presented later in his course of illness with anemia and did not have any other paraneoplastic syndrome. Pure red cell aplasia is usually associated with spindle cell type morphology (type A); however, this patient had type B (lymphocyte-rich) morphology [3].

Pure red cell aplasia can be divided into congenital and acquired. Acquired pure red cell aplasia (PRCA) causes profound anemia, characterized by reticulocytopenia and the absence of erythroid precursor cells in bone marrow. Acquired PRCA can be divided into primary and secondary. Thymoma is associated with secondary acquired PRCA [4].

PRCA in thymoma is postulated to be due to serum thymic factor, which is produced by thymoma. Serum thymic factor induces suppressor activity of T cells, which then inhibit erythroid precursors, leading to red cell aplasia. Other postulated mechanisms are thymic production of IgG which releases cytotoxic or suppressive cytokines that inhibit erythroid colony formation [5]. PRCA should be suspected when a patient comes with severe anemia with inappropriately low reticulocytes. After ruling out common causes for marrow failure, a bone marrow biopsy shall be performed for diagnostic purposes.

As thymoma-associated PRCA is an autoimmune-mediated disease, the treatment approach is mainly via targeting the autoimmune pathway and is divided into surgical and medical treatment. The best modality of treatment is complete resection of thymoma, as it leads to reduction in serum thymic factors, leading to reduction in erythroid inhibition [6,7]. However, one case report suggests complete resection of thymoma which is insufficient for the treatment of PRCA and PRCA could occur even after resection of thymoma [8,9].

Medical management of thymoma associated with PRCA includes immunosuppressive therapy, such as cyclosporine A [9,10]. Cyclosporine is effective in maintaining complete remission in patients with PRCA as reported by Hirokawa et al. [10]. None of the 20 patients treated with cyclosporine in that series relapsed in the 18 months observation period. However, cyclosporine fails to induce lifelong remission without maintenance therapy, as relapses are reported once cyclosporine is stopped [9]. The median dose of cyclosporine A used by Hirokawa et al. was 4.6 mg/kg/day for induction. Apart from cyclosporine A, other immunosuppressives such as corticosteroids and cyclophosphamide are also reported in treating PRCA. However, the clinical response seen in these groups of patients is not as robust as cyclosporine A group.

During maintenance treatment with cyclosporine, regular monitoring is required for drug toxicity and adverse effects. We performed renal function tests, electrolytes, and cyclosporine trough levels monthly for our patient at initiation and every four months thereafter.

## Conclusions

Pure red cell aplasia is a rare complication of thymoma. It should be suspected in all patients presenting with normochromic normocytic anemia with marked reticulocytopenia. Maintenance cyclosporine remains an effective medical management of thymoma-associated PRCA, leading to remission of anemia and improvement of reticulocyte counts.

## References

[1] Detterbeck FC, Zeeshan A (2013). Thymoma: current diagnosis and treatment. Chin Med J (Engl).

[2] Zhao J, Bhatnagar V, Ding L (2020). A systematic review of paraneoplastic syndromes associated with thymoma: treatment modalities, recurrence, and outcomes in resected cases. J Thorac Cardiovasc Surg.

[3] Means RT (2022). Acquired pure red cell aplasia in adults. UpToDate.

[4] Meneshian A, Olivier KR, Molina JR (2021). Clinical presentation and management of thymoma and thymic carcinoma. UpToDate.

[5] Lahiri TK, Agrawal D, Agrawal K, Shukla J (2002). Pure red cell aplasia associated with thymoma. Indian J Chest Dis Allied Sci.

[6] Lin CS, Yu YB, Hsu HS, Chou TY, Hsu WH, Huang BS (2009). Pure red cell aplasia and hypogammaglobulinemia in a patient with thymoma. J Chin Med Assoc.

[7] Jiang D, Wu M (2010). Thymectomy resulted in complete remission of pure red cell aplasia when associated with thymoma. Thorac Cardiovasc Surg.

[8] Thompson CA, Steensma DP (2006). Pure red cell aplasia associated with thymoma: clinical insights from a 50-year single-institution experience. Br J Haematol.

[9] Moriyama S, Yano M, Haneda H (2018). Pure red cell aplasia associated with thymoma: a report of a single-center experience. J Thorac Dis.

[10] Hirokawa M, Sawada K, Fujishima N (2008). Long-term response and outcome following immunosuppressive therapy in thymoma-associated pure red cell aplasia: a nationwide cohort study in Japan by the PRCA collaborative study group. Haematologica.

